# Oromandibular Dystonia: A Clinical Examination of 2,020 Cases

**DOI:** 10.3389/fneur.2021.700714

**Published:** 2021-09-16

**Authors:** Laura M. Scorr, Stewart A. Factor, Sahyli Perez Parra, Rachel Kaye, Randal C. Paniello, Scott A. Norris, Joel S. Perlmutter, Tobias Bäumer, Tatiana Usnich, Brian D. Berman, Marie Mailly, Emmanuel Roze, Marie Vidailhet, Joseph Jankovic, Mark S. LeDoux, Richard Barbano, Florence C. F. Chang, Victor S. C. Fung, Sarah Pirio Richardson, Andrew Blitzer, H. A. Jinnah

**Affiliations:** ^1^Department of Neurology, Emory University, Atlanta, GA, United States; ^2^Department of Otolaryngology, Rutgers University, Newark, NJ, United States; ^3^Department of Neurology, Washington University School of Medicine, St. Louis, MO, United States; ^4^Department of Neurology, Institute of Systems Motor Science, Universität of Lübeck, Lübeck, Germany; ^5^Department of Neurology, University of Colorado Anschutz Medical Campus, Aurora, CO, United States; ^6^Department of ENT and Head and Neck Surgery, Fondation Adolphe de Rothschild, Paris, France; ^7^Department of Neurology, Hôpital de la Pitié Salpétrière, Assistance Publique-Hôpitaux de Paris, Paris, France; ^8^Baylor St. Luke's Medical Center, Houston, TX, United States; ^9^Veracity Neuroscience LLC, Memphis, TN, United States; ^10^Department of Neurology, University of Memphis, Memphis, TN, United States; ^11^Department of Neurology, University of Rochester, Rochester, NY, United States; ^12^Department of Neurology, Westmead Hospital and Sydney Medical School, University of Sydney, Sydney, NSW, Australia; ^13^Department of Neurology, University of New Mexico Health Sciences Center, Albuquerque, NM, United States; ^14^Head and Neck Surgical Group, New York, NY, United States

**Keywords:** dystonia, jaw, tongue, treatment, botulinum (neuro)toxin

## Abstract

**Objective:** The goal of this study is to better characterize the phenotypic heterogeneity of oromandibular dystonia (OMD) for the purpose of facilitating early diagnosis.

**Methods:** First, we provide a comprehensive summary of the literature encompassing 1,121 cases. Next, we describe the clinical features of 727 OMD subjects enrolled by the Dystonia Coalition (DC), an international multicenter cohort. Finally, we summarize clinical features and treatment outcomes from cross-sectional analysis of 172 OMD subjects from two expert centers.

**Results:** In all cohorts, typical age at onset was in the 50s and 70% of cases were female. The Dystonia Coalition cohort revealed perioral musculature was involved most commonly (85%), followed by jaw (61%) and tongue (17%). OMD more commonly appeared as part of a segmental dystonia (43%), and less commonly focal (39%) or generalized (10%). OMD was found to be associated with impaired quality of life, independent of disease severity. On average, social anxiety (LSA score: 33 ± 28) was more common than depression (BDI II score: 9.7 ± 7.8). In the expert center cohorts, botulinum toxin injections improved symptom severity by more than 50% in ~80% of subjects, regardless of etiology.

**Conclusions:** This comprehensive description of OMD cases has revealed novel insights into the most common OMD phenotypes, pattern of dystonia distribution, associated psychiatric disturbances, and effect on QoL. We hope these findings will improve clinical recognition to aid in timely diagnosis and inform treatment strategies.

## Introduction

Dystonia is a movement disorder characterized by sustained or intermittent muscle contractions causing abnormal, often repetitive movements, abnormal postures, or both (1). The clinical presentations of oromandibular dystonia (OMD) include varying combinations of abnormal jaw, tongue, or lower face movements (2). OMD symptoms may be task specific, triggered by speech or eating, or can be present at rest. OMD is particularly disabling because it interferes with the ability to eat and speak, and may be associated with marked discomfort.

Idiopathic focal OMD is rare, representing 3–5% of all dystonias. Estimated incidence is 3.3/1,000,000 per year and estimated prevalence is 68.9/1,000,000 (3). OMD is often unrecognized, leading to a delay in diagnosis and treatment (4, 5). The average time from onset of symptoms to diagnosis in the most common forms of dystonia is 6 years, and this delay is believed to be even longer in OMD (6, 7). Also due to its rarity, much of our understanding is based on case reports or relatively small series. Some of these focused on idiopathic cases, others included acquired forms (8), and some included a high representation of inherited disorders with OMD, such as X-linked dystonia parkinsonism (9, 10). The descriptions of OMD from these centers are quite varied. For example, some expert centers recommend specific strategies for therapy with Botulinum toxin (BoNT) (9, 11–13), while others do not recommend it at all (14, 15). Some suggest that response of tardive and idiopathic forms of OMD to BoNT is similar, but large studies are lacking (16).

The purpose of this study is to better describe the clinical heterogeneity of OMD using a three-tiered approach. First, we provide a comprehensive summary of the largest prior studies of OMD, encompassing 1,121 total cases in 27 separate reports. Second, we describe the clinical features of OMD from the Dystonia Coalition (DC), a methodical international multicenter study of all types of dystonia (17), 727 of whom had OMD. Third, we provide details on the treatment response of 172 cases from two expert centers and investigate whether responses vary by etiology. The collection of different types of information from very different sources provides a comprehensive picture of this very complex disorder. By providing a more comprehensive description of OMD, we hope to improve clinical recognition to facilitate timely diagnosis and treatment.

## Materials and Methods

### Literature Review

The PubMed database was queried from January 1989 through January 2021 for reports using the keywords “oromandibular dystonia,” “jaw dystonia,” “Meige syndrome,” and “lingual dystonia.” Other reports were found through the article bibliographies. Only papers published in English with human subject data were included. Case reports of <5 subjects were excluded. Reviews that did not provide cohort or case demographic or clinical data were excluded. Series that described dystonia due to peripheral injury were excluded. Lower facial twisting typical of functional (psychogenic) dystonia were excluded due to diagnostic uncertainty. However, other acquired forms of OMD (e.g., tardive syndromes) were included because the majority of prior reports combined etiologies and it was not feasible to reliably distinguish tardive from idiopathic cases. Likewise, cases with task specific musician's dystonia were included because they were often described together with idiopathic cases. For this review, OMD was defined as dystonia affecting jaw, tongue and/or lower face. Bruxism has mixed pathophysiology including mechanical alignment issues, dental problems, disturbed sleep physiology, and peripheral nervous system pathology (18). Subjects with bruxism were only included if concurrently diagnosed with OMD. Subjects with temporomandibular joint disorders who did not clearly meet criteria for OMD were excluded (19, 20). Data extracted from published case series included patient demographics and dystonia etiology, clinical features, and treatment response. For a subset of the literature for which case level data was available, additional data was extracted on the frequency of phenotypic subtypes (jaw opening, jaw closing, jaw deviation, tongue, and face involvement).

### Dystonia Coalition Cohort

Data from the DC database were collected and analyzed for 727 OMD subjects enrolled across 26 international sites from 2011 to 2019 (http://clinicaltrials.gov/show/NCT01373424) (17). To be included in the DC database subjects had to be diagnosed with isolated dystonia of any type (focal, segmental, multifocal, generalized). For the current study of OMD, subjects were required to have dystonia in the jaw, tongue, and/or perioral region. Exclusion criteria included any evidence of acquired dystonia, significant medical or neurologic conditions that preclude completing the examination, or other significant condition that would confound diagnosis or evaluation. We defined focal OMD as involvement of the lower face, jaw or tongue. Segmental dystonia was diagnosed in subjects with dystonia in contiguous body regions such as the upper face, neck, or larynx.

Subjects completed a 26-item questionnaire describing demographics and characteristics of their dystonia. A neurologist specializing in movement disorders evaluated each participant to determine distribution and severity of dystonia as measured on the Global Dystonia Rating Scale (GDRS) (21). The GDRS is a Likert-like scale with which dystonia is rated from 0 (absent) to 10 (maximum severity). Subjects were also queried about any sensory trick (also known as geste antagoniste or alleviating maneuver) (22) and prior treatments utilized. Clinical features of interest included distribution of dystonia (focal, segmental, general), areas affected (jaw, tongue, lower face), and treatments utilized. Some subjects also completed the SF 36-Item Health Survey assessing quality of life (QOL), Beck Depression Inventory II scale (BDI), and Leibowitz Social Anxiety scale (LSA). These data were collected only for subjects with onset <5 years to limit recall bias.

### Expert Center Cohorts

Data were collected from two centers with expertise in OMD. Retrospective chart review identified a total 116 subjects evaluated at the Emory University Movement Disorders Clinic (EMDC) between 2015 and 2019, and 56 subjects evaluated in Head and Neck Surgical Group (HNSG) and the NY Center for Voice and Swallowing Disorders, all of whom were enrolled. EMDC is staffed by movement disorders neurologists, whereas HNSG is staffed by otolaryngologists, capturing two of the subspecialty fields that most commonly treat OMD. The primary inclusion criterion was idiopathic or acquired OMD, diagnosed as described previously. Exclusion criteria were loss to follow up or other significant condition that would confound diagnosis or treatment outcomes. Demographic and clinical features including age at onset, sex, race, distribution of dystonia, and areas affected were extracted from retrospective chart review. Rating scale data, QOL, depression, and social anxiety scores were not available for these subjects. However, additional information was collected on BoNT treatment.

### Data Analysis

Analyses were completed separately for each of the four cohorts (Literature review, DC, EMDC, and HNSG) due to the different types of data that were available for each. Although duplicate reporting of the same case cannot be entirely excluded, every effort was made to avoid including the same case more than once. The literature review summarizes cases from very different geographical locations, often from different countries, and therefore is not likely to contain many duplicates. The Dystonia Coalition tracks individual subjects and conducts DNA fingerprinting, so enrollment of the same case more than once can be identified and excluded. Patient identifiers were available for the expert centers, so that duplicate reporting of the same case could be definitively excluded.

Descriptive analyses for demographics and clinical characteristics were completed. For the expert center cohorts with detailed treatment data, additional descriptive analysis was performed for therapies employed and response to treatment. To evaluate whether treatment response varied by etiology, ANOVA was utilized. Within the DC cohort, additional descriptive analyses were performed for BDI, LSA, and SF-36 scores. Univariate linear regression was performed to estimate the association between QOL and demographic/clinical characteristics. A multivariate linear regression model was constructed accounting for age and sex, assessing QOL. Distribution of dystonia (focal, segmental, hemidystonia, and generalized) was also accounted for as a marker of severity. A small amount of missing data was identified in the DC dataset utilized for the regression analysis (<0.4%) and was treated as missing at random. All data analysis was performed with SAS version 9.4.

The study was approved by the internal review boards (IRBs) of all participating clinical sites. All subjects gave written consent for participation following the principles of the Declaration of Helsinki.

### Data Availability

All data from the DC database are available by request from the DC. Data on the expert center cohorts and extracted from literature review are available by request from the corresponding author.

## Results

### Review of Published Cases

A comprehensive literature query returned 27 papers meeting inclusion criteria. These papers describe 1,121 cases including idiopathic and acquired OMD (Table 1). The reports were generally of single center cohorts for the purpose of defining clinical features and treatment response. Only 6 described cohorts >50 subjects. Among these series, 68% of subjects were female and the mean age of onset was 52. Etiologies varied by report, but primarily included idiopathic and tardive cases. Although it is traditional to group OMD into specific subtypes (jaw opening, jaw closing, tongue, lower face), descriptions of clinical characteristics varied considerably, with some authors defining each case by the predominant feature and many describing a mixed picture. Among the 389 cases described by the predominant feature, 45% were jaw closing, 31% jaw opening, 24% mixed, and 1% lingual. Only three of 27 studies noted presence of lower facial or perioral involvement, and none defined this as the predominant feature. The majority of reports note that subjects received a trial of BoNT. Variable measurements of response were used in the literature, therefore Table 1 shows only the frequency of cases reporting some level of response. Some centers did not report on BoNT treatment and/or outcome, or treated some subjects exclusively with oral pharmacotherapy. However, the majority of reports describing BoNT note return for subsequent injection and subjective improvement in symptoms and/or QOL. No cases of OMD remission were described in the manuscripts reviewed.

**Table 1:** Clinical characteristics of 1,121 published cases of oromandibular dystonia.

**References**	***n***	**Etiology**	Clinical characteristics^*^	**BoNT trial (*n*)**	Percent of cases reporting BoNT response^**^
Blitzer et al. (23)	20	NR^+^	NR	Yes (20)	95%
Jankovic et al. (24)	62	NR	JC (NR), JO (NR)	Yes (62)	73%
Hermanowicz and Truong (25)	5	NR	JO (20%), JC (80%), L (20%)	Yes (5)	100%
Van den Bergh et al. (26)	5	NR	JO (80%), L (20%)	Yes (5)	100%
Sankhla et al. (27)	21	NR	NR	Yes (21)	68%
Tan and Jankovic (28)	162	Idiopathic (63%), Tardive (23%), Other (14%)	JO (22%), JC (53%), M (24%)	Yes (162)	68%
Tan and Jankovic (16)	116	Idiopathic (79%) Tardive (21%)	JO (23%), JC (49%), M (28%), L (20%)	Yes (116)	100%
Bhattacharyya et al. (29)	5	NR	NR	Yes (5)	100%
Adler et al. (30)	5	Idiopathic (100%)	JO (40%), JC (40%), M (20%)	Yes (5)	NR
Lo et al. (31)	5	Post-stroke (100%)	JC (100%)	Yes (5)	33%
Wan et al. (32)	12	NR	NR	Yes (12)	88%
Singer and Papapetropoulos (33)	23	NR	JO (52%), JC (48%)	Yes (23)	65%
Lee (34)	6	NR	JC (50%), L (50%), P (67%)	Yes (2)	100%
Rosales et al. (9)	49	X-linked dystonia-parkinsonism	JO (65%), JC (24%), M (12%)	Yes (49)	100%
Merz et al. (35)	30	Idiopathic (83%)Other (17%)	JO (10%), JC (23%), M (33%), L (10%)	Yes (30)	100%
Esper et al. (8)	17	Idiopathic (47%) Tardive (41%) Degenerative (6%) Post-infectious (6%)	JO (53%), JC (18%), M (18%), L (100%)	Yes (9)	78%
Teive et al. (36)	5	Wilson's disease (100%)	JO (100%)	Yes (5)	100%
Sinclair et al. (37)	59	Idiopathic (91%)Tardive (9%)	JO (36%), JC (48%) M (16%), L (17%)	Yes (59)	100%
Bakke et al. (38)	21	NR	JO (19%), JC (5%), M (43%)	N R	NR
Termsarasab et al. (14)	41	Idiopathic (100%)	M (63%), L (27%), P (5%)	Yes (18)	12%
Moscovich et al. (39)	8	NR	M (100%)	Yes (8)	100%
Teemul et al. (40)	6	Idiopathic (83%) Tardive (17%)	M (100%)	Yes (6)	83%
Nastasi et al. (41)	30	Idiopathic (73%) Tardive (13%)	JO (53%), JC (17%), M (7%), L (100%), P (27%)	Yes (30)	87%
Kreisler et al. (42)	14	NR	JO (21%), JC (36%), M (7%), P (29%)	Yes (7)	(All returned for repeat injections)
Slaim et al. (43)	240	Idiopathic (71%) Tardive (13%) Other (16%)	JO (62%), JC (20%), M (27%), L (27%)	No	NR
Scorr et al. (11)	18	Idiopathic (83%) Tardive (17%)	JO (50%), JC (17%), M (33%), L (33%),	Yes (18)	100%
Yoshida (12)	136	Tardive (31%)	M (20%), L (100%)	Yes (136)	NR

* *Jaw Opening (JO), Jaw Closing (JC), Mixed Jaw Phenomenology (M), Lingual (L), Perioral (P)*.

+ *Not reported (NR)*.

** *Percentage does not indicate level of therapeutic effect, but percentage of cases reporting some therapeutic effect*.

### DC Cohort

Table 2 presents a cross-sectional analysis of demographics and clinical features for subjects with idiopathic OMD enrolled in the DC database. Among the 727 cases, 70% were female and the mean age at onset was 50 ± 16 (mean ± SD) years. In this cohort, 87% of subjects identified as White. The distribution of dystonia was most commonly segmental (43%), and less commonly focal (39%) or generalized (10%). Sixty-one percent had involvement of jaw, 85% had involvement of lower face, and 17% had involvement of tongue. GDRS severity scores averaged 3 ± 2, for the lower face 4 ± 2 for the jaw and tongue, with average total scores of 16 ± 13. Among the subjects, 32% reported having received BoNT treatment, but treatment details were not available. Concurrent dystonia in other regions of the body did not increase the chance that OMD patients received BoNT therapy (*p* = 0.53).

**Table 2:** Clinical characteristics and treatment response of oromandibular dystonia subjects in the Dystonia Coalition database, EMDC Cohort, and Head and Neck Surgical Group (HNSG) Cohort.

**Characteristics**	**Dystonia coalition** ***N*** **= 727** ***n*** **(%)/mean ± SD**	**EMDC Cohort** ***N*** **= 116** ***n*** **(%)/mean ± SD**	**HNSG Cohort** ***N*** **= 56** ***n*** **(%)/mean ± SD**
**Age of dystonia onset**	50 ± 16	54 ± 15	50 ± 14
Focal	53 ± 12	59 ± 12	NR^*^
Segmental	50 ± 14	53 ± 14	NR
Generalized	26 ± 20	29 ± 22	NR
**Sex**			
Male	217 (30%)	35 (30%)	7 (30%)
Female	510 (70%)	81 (70%)	49 (70%)
**Race**			
White	634 (87%)	76 (66%)	20 (36%)
Black	57 (8%)	27 (23%)	2 (4%)
Other	30 (4%)	11 (9%)	4 (7%)
Unknown	6 (1%)	2 (2%)	30 (53%)
**Etiology**			
Idiopathic	727 (100%)	75 (65%)	46 (82%)
Tardive	NR	25 (22%)	10 (18%)
Degenerative	NR	11 (9%)	NR
Stroke induced	NR	3 (2%)	NR
Genetic	NR	2 (2%)	NR
**Distribution**			
Focal	284 (39%)	53 (46%)	46 (82%)
Segmental	315 (43%)	57 (49%)	9 (16%)
Multifocal	46 (6%)	NR	NR
Hemi-dystonia	5 (1%)	NR	NR
Generalized	74 (10%)	6 (5%)	1 (2%)
**Areas affected**			
Lower face	620 (85%)	31 (29%)	3 (5%)
Jaw	440 (61%)		
Opening	NR	40 (36%)	22 (39%)
Closing	NR	61 (56%)	12 (21%)
Deviation	NR	17 (16%)	2 (4%)
Mixed	NR	NR	20 (36%)
Tongue	123 (17%)	40 (36%)	7 (12%)
**GDRS severity**			
Total	16 ± 13	NR	NR
Lower face	3 ± 2	NR	NR
Jaw and tongue	4 ± 2	NR	NR
**BoNT**			
Treated	234 (32%)	115 (99%)	56 (100%)
Dose (Onabotulinum toxin A equivalent units)	NR	102 (72)	73 (40)
EMG usage	NR	101 (88%)	NR
**Toxin Response**			
Good (75–100%)	NR	48 (45%)	48 (84%)
Moderate (50–74%)	NR	24 (23%)	3 (5%)
Partial (25–49%)	NR	2 (9%)	0
Minimal (1–24%)	NR	19 (18%)	1 (2%)
Not reported	NR	1 (1%)	4 (8%)

* *Not Reported (NR)*.

Patient reported disability was measured by the SF-36 (Table 3), which was transformed such that a score of zero is equivalent to maximum disability and a score of 100 is equivalent to no disability. Average scores represent disability in all domains, particularly in mental health (26 ± 20), physical role (50 ± 43), emotional role (50 ± 14), and vitality (53 ± 21). Mood was evaluated with BDI, where total scores >13 are indicative of depression (44). The average BDI score among subjects with OMD was 9.7 ± 7.8, and 34% had scores indicative of depression. Anxiety was assessed using the LSA score, where scores >30 are consistent with social anxiety (45). The average LSA score among subjects with OMD was 33 ± 28, and 44% had scores indicative of social anxiety.

**Table 3:** Depression, anxiety, and QOL in oromandibular dystonia subjects enrolled in the dystonia coalition natural history study.

**Characteristics**	**Dystonia coalition natural history study** ***N*** **= 368** **Mean ± SD**
**SF-36 sub-scores**	
Physical function	71 ± 27
Role physical	50 ± 43
Body pain	63 ± 27
General health	62 ± 22
Vitality	53 ± 21
Social function	66 ± 29
Role emotional	50 ± 14
Mental health	26 ± 20
**LSA score**	33 ± 28
**BDI score**	10 ± 8

Univariate linear regression revealed age, sex, distribution, and severity of dystonia as measured by GDRS were not significantly associated with total QOL score. Subjects identifying as Black had QOL scores that were worse than subjects identifying as White, indicating significantly worse physical QOL (β = −10.25, *p* = 0.02). Mental QOL improved 0.34 points for each year older a patient was, indicating worse mental QOL for younger subjects with OMD (β = 0.34, *p* < 0.01). GDRS severity score was associated with worsened mental QOL, and each point higher GDRS score was associated with a 0.6-point lower mental component QOL score (β = −0.60, *p* < 0.01). Each point higher on the BDI scale for depression was associated with a 1-point lower mental and physical component QOL score (*p* < 0.01). Each point higher on the LSA scale for anxiety was associated with a 0.28-point and 0.14-point lower score for mental and physical component QOL score, respectively (*p* < 0.01). Reported exposure to BoNT therapy was not associated with QOL scores. These findings underscore the negative effect of OMD on QOL as well as the significance of comorbid depression and social anxiety in this population.

### Expert Center Cohorts

#### Emory Movement Disorders Clinic

A retrospective analysis was performed for the cohort of 116 OMD subjects evaluated and treated at EMDC within the last 5 years, who were not already enrolled in the DC (Table 2). Among these cases, 70% were female. The majority of subjects had segmental (49%) and focal (46%) distributions, as compared to generalized (5%). The most frequently affected area was jaw (63%), though involvement of lower face (29%) and tongue (36%) were common. The most common jaw movement was closing (56%), followed by opening (36%), or deviation (17%). Of the subjects seen at least twice, 99% received BoNT. The average BoNT-A equivalent unit dosage was 102 ± 72 u. EMG was used in 88% of cases to confirm injection placement. Routine clinical practice at EMDC is to record improvement according to percentiles, with 0% being no improvement and 100% being complete relief of symptoms. Subjective improvement ranging from 50 to 100% was reported by 68% of cases.

Among all cases, nine had remission (Table 4), all were women. Three of these were White, three Black, one Asian, and two of unknown race. The mean age at onset was 55 ± 12 years, and diagnosis was 32 ± 35 months after onset. On average, 13 ± 10 BoNT sessions were provided prior to remission. The mean dose used in the last visit prior to remission was 92 ± 86 units. The mean duration of symptoms was 7 ± 5 years and mean duration of remission was 20 ± 17 months at the time of chart review. Seven subjects had idiopathic and two had tardive OMD. Eight subjects had jaw opening dystonia, one patient had jaw deviation and one case presented a mixed form (jaw opening and jaw deviation). Lingual dystonia was present in six subjects.

**Table 4:** Characteristics of remission in oromandibular dystonia cases.

**Characteristics**	**EMDC remission cases** ***N*** **= 9** ***N*** **(%)/mean ± SD**
**Age of dystonia onset**	55 ± 12
**Sex**	
Female	9 (100%)
**Race**	
White	3 (33%)
Black	3 (33%)
Asian	1 (11%)
Unknown	2 (22%)
**Etiology**	
Idiopathic	7 (78%)
Tardive	2 (22%)
**Distribution**	
Focal	5 (56%)
Segmental	4 (44%)
**Areas Affected**	
Jaw	
Opening	8 (89%)
Closing	0
Deviation	1 (11%)
Mixed	1 (11%)
Tongue	6 (67%)
**BoNT**	
Treated	9 (100%)
Dose (Onabotulinum toxin A equivalent units)^*^	92 (86)
**Duration of symptoms** (years)	7 ± 5

* *Dose conversion ratio of AbobotulinumtoxinA to OnabotulinumtoxinA was 2.5:1*.

#### Head and Neck Surgical Group Cohort

A retrospective analysis was performed on 56 OMD subjects evaluated at HNSG within the last 5 years (Table 2). Among these cases, 70% were female. The majority had focal dystonia (82%), as compared to segmental (15%) and generalized (2%). The most common area affected was the jaw and presentations included jaw opening (39%), jaw closing (21%), jaw deviation (4%), and mixed phenomenology (36%). Additional lingual dystonia was present in 13%. All subjects received BoNT and the average BoNT-A equivalent dosage was 73 ± 40 u. Patients reported improvement in pain and/or function as good in 84% of cases.

#### Evaluation of Differences in Clinical Features and Treatment Response by Etiology

To evaluate whether clinical characteristics and BoNT treatment response varied by etiology, the data from EMDC and the HNSG were pooled for analysis. Etiology of OMD was categorized as idiopathic, tardive, or other (degenerative, post-stroke, related to a genetic syndrome such as Wilson's disease or Pantothenate kinase-associated neurodegeneration. Clinical characteristics and treatment responses were investigated to determine whether they were a function of etiology (Table 5). Demographic features including age of onset and sex distribution did not vary significantly by etiology. Features of lingual dystonia were more common among tardive cases (*p* = 0.03), but there was no significant difference in the occurrence of other phenomenology's by etiology. In contrast to previous reports (46, 47), total toxin dose required (*p* = 0.78) and toxin response (*p* = 0.56) did not vary by etiology.

**Table 5:** Variation in clinical characteristics and treatment response of oromandibular dystonia by etiology in expert center cases.

**Characteristics**	**Idiopathic** ***N*** **= 121** ***n*** **(%)/mean ± SD**	**Tardive** ***N*** **= 36** ***n*** **(%)/mean ± SD**	**Other** ***N*** **= 16** ***n*** **(%)/mean ± SD**	***p*** **-value**
**Age at dystonia onset**	54 ± 13	54 ± 15	46 ± 23	0.97
**Sex**				0.54
Male	36 (30%)	9 (30%)	6 (40%)	
Female	85 (70%)	27 (74%)	10 (60%)	
**Areas affected**				
Lower face	33 (27%)	8 (21%)	3 (20%)	0.76
Jaw				
Opening	59 (49%)	14 (40%)	4 (26%)	0.15
Closing	64 (53%)	21 (57%)	13 (80%)	0.09
Deviation	17 (14%)	4 (11%)	1 (5%)	0.56
Tongue	33 (27%)	15 (43%)	2 (10%)	0.03
**BoNT**				
Dose (Onabotulinum toxin A equivalent units)	88 ± 64	73 ± 46	90 ± 75	0.48
Toxin response				0.56
None	5 (4%)	0	0	
>50% improvement	96 (79%)	27 (76%)	13 (80%)	

## Discussion

This study provides the largest and most comprehensive summary of the phenotypic heterogeneity of OMD, to guide improved recognition of this rare and particularly disabling subtype of dystonia. It encompasses a total of 2,020 cases, including 1,121 cases reported in the world's literature, 727 new cases from an international multicenter cohort, and 172 new cases treated at 2 expert centers. Each of these three sources of information regarding OMD has different strengths and weaknesses. Despite the very different sources of information, the overall results provide a remarkably consistent picture. Like other focal dystonias, OMD tends to emerge in adults in the early 50s (48, 49). Like other focal dystonias, it is more common in women (50). Despite these similarities, each of the three sources of information also provides novel insights into the varied nature of OMD.

### Strengths and Weaknesses of Different Sources

Combining data across very different sources is valuable for identifying the most consistent findings. However, there also are some limitations with including information from different sources. One is the varied inclusion criteria and methods of assessment and reporting make it difficult to merge all data into a single analysis. Another weakness is the possibility of duplication, as it is always difficult to be sure that some cases are not reported more than once. For the literature review, this is unlikely, because most of the reports come from geographically distant areas, and often different countries. For the DC, a standard procedure is used to prevent duplicate recruitment of cases. For the expert centers, the availability of patient identifiers allowed us to exclude any duplications, and we were careful to avoid any subjects recruited by these centers for the Dystonia Coalition. Additionally, although the amount of missing data was very small (<0.4%), we cannot rule out that non-random patterns of missingness in the DC data may have introduced some biases (likely of small magnitude) in the results.

The literature provides the largest source of information on OMD, with a total of 1,121 cases. However, the approach to diagnosis and evaluation of OMD are also the most varied, driven by varying habits used at different centers, and leading to significant differences in findings. For example, some centers reported mostly idiopathic OMD, while others focused on tardive OMD or specific inherited subtypes such as X-linked dystonia-parkinsonism. Some centers described cases according to the most affected region (tongue, jaw, lower face), while others provided more details such as the type of jaw (e.g., closing, opening, mixed) or tongue (e.g., protrusion, retraction, lateral deviation) movements. Strategies and outcomes for treatment were perhaps the most varied.

Data from the two expert centers provide new information for a large number of new OMD cases (*n* = 172), along with detailed information regarding treatment strategies. These data also revealed several cases of long-lasting remissions, a novel finding not apparent in the literature. Unlike with cervical dystonia (51), remission in OMD occurred later in the disease course, after mean disease duration of 6.9 years. The expert center data were subject to some of the same limitations described for the published literature. Both of these centers used somewhat different approaches for diagnosis, evaluation, treatment, and recording of treatment outcomes. Since the data from both centers came from documentation regarding clinical care, certain details were not always available, such as methodical assessment of severity using rating scales, or detailed assessment of body regions outside of the oromandibular region.

The data from the DC also provides new information for a large number of OMD cases (*n* = 727). Since multiple centers used the same protocols for evaluation, these data reflect the most consistent information from the largest number of different centers (17). These data also include quantitative rating scale data for all body regions. Additionally, this cohort provided novel insights into frequent psychiatric symptoms associated with OMD, and their significant impact on QOL. However, the DC included only isolated OMD, while the literature and expert centers included mixed types of OMD. Further, the DC did not collect data regarding treatment responses, or specific types of movement abnormalities of the jaw (e.g., closing, opening, mixed) or tongue (e.g., protrusion, retraction, lateral deviation).

### Similarities and Differences Among Different Sources

As noted above, the three sources of data provided remarkably consistent information for certain features of OMD. They also suggested some differences. For example, the literature described most cases of OMD as a focal dystonia, although methods for assessment of other body regions were not often reported (35). On the other hand, data from the DC and one of the expert centers suggest OMD is more often part of a broader segmental pattern of dystonia. Similarly, involvement of the lower face is uncommonly reported among cases in the literature, but very common among cases in the DC, most likely because of the methodical assessment of all body regions. Among studies that focused on jaw dystonia, it seems likely there is a high probability that less seriously affected regions like the lower face may not be reported.

In addition to body distribution of dystonia, severity is also an important aspect of OMD. Unfortunately, severity is difficult to compare across the groups because of markedly different methods used for assessment. Severity assessments included clinician-rated dystonia scales, patient reported outcomes such as the Oromandibular Dystonia Questionnaire (OMDQ-25) (35), Likert-like scales, or clinical impression. In the DC cohort where severity was systematically assessed with the GDRS, total scores were 16 ± 13, with much lower severity in the lower face (3 ± 2) or jaw/tongue (4 ± 2). Despite the low GDRS scores for OMD, the marked impact on QOL suggests that OMD is a particularly disabling form of dystonia. This discrepancy suggests the GDRS may not adequately capture OMD, perhaps because movements of the jaw and tongue are difficult to see, and that routine use of OMD-specific scales may be needed (52). Alternatively, it could imply that non-motor features have a greater impact on QOL in OMD, similar to other focal dystonias (53).

The DC included mostly idiopathic cases, while cases from the literature and the two expert centers also included acquired or combined dystonias. Nevertheless, overall results were remarkably similar across these sources. Although some reports suggest that dystonia in the jaw and tongue is a red flag that should alert the clinician to an inherited metabolic disorder such as neuroacanthocytosis or X-linked dystonia-parkinsonism (47), results from the DC and expert centers indicate that OMD is also, not uncommonly, the result of idiopathic and tardive disease. It was possible to directly compare idiopathic and tardive OMD for the expert centers, and the only difference involved more significant tongue involvement in tardive cases.

A final area where there are significant differences in the literature relates to treatment. Some studies argue the response to oral therapy is strongest (14), while others recommend BoNT (9, 11, 16, 28). Of 23 manuscripts reporting treatment response, 11 reported 100% of cases improved, 10 reported >65% of cases improved, and only two reported <50% of cases improved (Table 1). Review of data from the expert centers provided more detailed information on responses to BoNT. In both centers, most subjects were treated with BoNT. Most reported >50% improvement. At both centers, <10% of subjects reported no improvement. Treatment responses were not significantly associated with etiology with 79% of idiopathic and 76% of tardive OMD patients reporting >50% improvement in symptoms. One limitation of treatment data is that outcomes are retrospective and based on subjective patient report, which is susceptible to bias. Another limitation may be the heterogeneity of possible pathogenic mechanisms among the cases analyzed, though analysis by etiology did not reveal significant differences in treatment response.

In addition to the many original reports of OMD summarized, there have also been several reviews and commentaries focusing on OMD (4, 5, 15, 54). Though these reviews focus on different aspects of OMD, they also describe a picture consistent with our report relating to age of onset, female predominance, and delayed diagnosis due to poor recognition of the varied phenotypes of OMD. Most experts agree that the varied phenotypes of OMD represent a clinical syndrome arising from multiple factors. A combination of genetic and environmental factors are thought to combine to reach a threshold for manifestation of clinical symptoms (54). Early symptoms of disease are varied and may be subtle, which is believed to contribute to delayed diagnosis may result in a higher actual prevalence than previously reported (4). In an effort to address the problem of efficient diagnosis, a group of Italian Movement Disorder experts formulated clinical diagnostic recommendations for oromandibular dystonia. Their proposed clinical diagnostic algorithm utilizes the consensus definition of dystonia and leverages presence of sensory trick vs. atypical features to suggest a diagnosis of OMD (5). Even when the diagnosis is certain, a number of reviews and commentaries have debated the best treatment strategy. Our study focused on BoNT treatments, because they are the treatment of first choice for most focal dystonias (55). Despite this, some have argued routine use cannot be established in the absence of large controlled studies to establish efficacy and safety (15). A hindrance to large clinical trials has been the rarity of this disorder, but it is possible that improved clinical recognition may make such trials more feasible. Although a number of alternative treatments have been proposed for OMD [e.g., pharmacotherapy, neurosurgical procedures including deep brain stimulation (56), muscle afferent block therapy, and acupuncture] (55), there were insufficient data in the literature reviewed to compare the efficacy of these alternative strategies. Further studies of treatment strategies are warranted.

## Conclusions

Each tier of analysis in this study revealed novel insights into the phenotypic heterogeneity of OMD. The frequency of OMD presenting as a feature of segmental dystonia underscores the importance of standardized clinical assessment of cases for dystonia in other body regions. Assessment may also benefit from utilization of disease specific rating scales (52) to more accurately measure severity and capture less prominent features, such as involvement of the perioral lower facial musculature. Additionally, it may be important to include screening for psychiatric disturbances, which were found to be major determinants of QOL in this population. Our findings suggest that BoNT injection is an effective treatment for the majority of patients, regardless of etiology. Prospective controlled trials may be useful to clarify the best treatment strategies. Future directions also include investigating the natural history of OMD to determine predictors of progression or remission for this particularly disabling form of dystonia.

## Data Availability Statement

The raw data supporting the conclusions of this article will be made available by the authors, without undue reservation.

## Ethics Statement

The study was approved by the internal review boards (IRBs) of all participating clinical sites, including the Emory Institutional Review Board. All subjects gave written consent for participation following the principles of the Declaration of Helsinki. The patients/participants provided their written informed consent to participate in this study.

## Author Contributions

LS, SF, and HJ: drafting/revision of the manuscript for content, including medical writing for content, major role in the acquisition of data, study concept or design, and analysis or interpretation of data. SPa: drafting/revision of the manuscript for content, including medical writing for content, major role in the acquisition of data, and analysis or interpretation of data. RK, RP, SN, JP, TB, TU, BB, MV, JJ, ML, RB, FC, VF, ER, and SPi: drafting/revision of the manuscript for content, including medical writing for content, and major role in the acquisition of data. MM: major role in the acquisition of data. AB: drafting/revision of the manuscript for content, including medical writing for content, major role in the acquisition of data, and study concept or design. All authors contributed to the article and approved the submitted version.

## Funding

This study was supported in part by the Dystonia Medical Research Fellowship Award (no award number) and in part by grants to the DC, a consortium of the Rare Diseases Clinical Research Network (RDCRN) that was supported by U54 TR001456 from the Office of Rare Diseases Research (ORDR) at the National Center for Advancing Clinical and Translational Sciences (NCATS) and U54 NS065701 and U54 NS116025 from the National Institute for Neurological Diseases and Stroke (NINDS). The Sartain Lanier Family Foundation as well as the Jean and Paul Amos Parkinson's Disease and Movement Disorders Program Endowment also provided support for this study (no award number).

## Conflict of Interest

HJ has active or recent grant support from the US government (National Institutes of Health), private philanthropic organizations (Cure Dystonia Now), and industry (Retrophin, Inc.; Revance Therapeutics, Inc.). HJ has also served on advisory boards or as a consultant for Allergan Inc., CoA Therapeutics, Cavion Therapeutics, EnePharmaceuticals, Ipsen, and Retrophin Inc. He has received honoraria or stipends for lectures or administrative work from the American Academy of Neurology, the American Neurological Association, the Dystonia Medical Research Foundation, the International Neurotoxin Society, and the International Parkinsons Disease and Movement Disorders Society. HJ serves on the Scientific Advisory Boards for several private foundations including the Benign Essential Blepharospasm Research Foundation, Cure Dystonia Now, the Dystonia Medical Research Foundation, the Tourette Association of America, and Tyler's Hope for a Cure. He also is principle investigator for the Dystonia Coalition, which has received the majority of its support through the NIH (grants NS116025, NS065701 from the National Institutes of Neurological Disorders and Stroke TR 001456 from the Office of Rare Diseases Research at the National Center for Advancing Translational Sciences). The Dystonia Coalition has received additional material or administrative support from industry sponsors (Allergan Inc. and Merz Pharmaceuticals) as well as private foundations (The Benign Essential Blepharospasm Foundation, Cure Dystonia Now, the Dystonia Medical Research Foundation, and the National Spasmodic Dysphonia Association). SF has received honoraria from Lundbeck, Sunovion, Biogen, Acadia, Impel, Acorda, and CereSpir. He has received grant support from Medtronics, Boston Scientific, Biohaven, Impax, Lilly, US World Meds, Sunovion Therapeutics, Vaccinex, Voyager, Jazz Pharmaceuticals, CHDI Foundation, Michael J. Fox Foundation, NIH (U10 NS077366), Parkinson Foundation. He has received royalties from Demos, Blackwell Futura, Springer for textbooks, Uptodate. SN receives grant support from NIH including NS107281 (JP), NS103957 (JP and TU), TR00135609 (HJ), and the Dystonia Medical Research Foundation. JP has received research funding from National Institutes of Health NS075321, NS103957, NS107281, NS092865, U10NS077384, NS097437, U54NS116025, U19 NS110456, AG050263, AG-64937, NS097799, NS075527, ES029524, NS109487, R61 AT010753 (NCATS, NINDS, NIA), Department of Defense (DOD W81XWH-217-1-0393), Michael J Fox Foundation, Barnes-Jewish Hospital Foundation (Elliot Stein Family Fund and Parkinson disease research fund), American Parkinson Disease Association (APDA) Advanced Research Center at Washington University, Greater St. Louis Chapter of the APDA, Paula and Rodger Riney Fund, Jo Oertli Fund, Huntington Disease Society of America, Murphy Fund, and CHDI. I have received honoraria from CHDI, Huntington Disease Study Group, Parkinson Study Group, Beth Israel Hospital (Harvard group), U Pennsylvania, Stanford U. He also is co-director for the Dystonia Coalition, which has received the majority of its support through the NIH (grants NS116025, NS065701 from the National Institutes of Neurological Disorders and Stroke TR 001456 from the Office of Rare Diseases Research at the National Center for Advancing Translational Sciences). JP serves as Director of Medical and Scientific Advisory Committee of the Dystonia Medical Research Foundation, Chair of the Scientific Advisory Committee of the Parkinson Study Group, Chair of the Standards Committee of the Huntington Study Group, member of the Scientific Advisory Board of the APDA, Chair of the Scientific and Publication Committee for ENROLL-HD, and member of the Education Committee of the Huntington Study Group. JP has provided medical legal consultation to Wood, Cooper and Peterson, LLC and to Simmons and Simmons LLP. TB is supported by the German Research Foundation (SFB 936 and FOR 2698), the German federal research ministry (BMBF) through the Dystonia Translational Research and Therapy Consortium (DysTract) (Grant No. 01GM1514A) and the Innovationsausschuss of the Gemeinsamer Bundesausschuss (Translate NAMSE, structural support for the Lübeck Center for Rare Diseases). He receives honoraria from Merz Pharmaceuticals, Allergan and Ipsen Pharma. He is member of the advisory boards of Merz Pharmaceuticals, Allergan and Ipsen Pharma and receives consultancies from Merz Pharmaceuticals and Allergan. ER served on scientific advisory boards for Orkyn, Aguettant, Merz-Pharma; received honoraria for speeches from Orkyn, Aguettant, Merz-Pharma, Everpharma, International Parkinson and Movement disorders Society; received research support from Merz-Pharma, Orkyn, Aguettant, Elivie, Ipsen, Everpharma, Fondation Desmarest, AMADYS, Fonds de Dotation Brou de Laurière, Agence Nationale de la Recherche; received travel grant from Vitalair, PEPS development, Aguettant, Merz-Pharma, Ipsen, Merck, Orkyn, Elivie, Adelia Medical, Dystonia Medical Research Foundation, International Parkinson and Movement disorders Society, European Academy of Neurology, International Association of Parkinsonism and Related Disorders. JJ has received research/training funding from AbbVie Inc; Acadia Pharmaceuticals; Allergan, Inc; Biotek; Cerevel Therapeutics; CHDI Foundation; Dystonia Coalition; Emalex Biosciences, Inc; F. Hoffmann-La Roche Ltd; Huntington Study Group; Medtronic Neuromodulation; Merz Pharmaceuticals; Michael J Fox Foundation for Parkinson Research; National Institutes of Health; Neuraly, Inc.; Neurocrine Biosciences; Parkinsons Foundation; Parkinson Study Group; Prilenia Therapeutics; Revance Therapeutics, Inc; Teva Pharmaceutical Industries Ltd. JJ has served as a consultant for Aeon BioPharma; Nuvelution Pharma, Inc; Teva Pharmaceutical Industries Ltd. JJ has received royalties from Cambridge; Elsevier; Medlink: Neurology; Lippincott Williams and Wilkins; Wiley-Blackwell. Editorial boards: Expert Review of Neurotherapeutics; Medlink; Neurology in Clinical Practice; The Botulinum Journal; PeerJ; Therapeutic Advances in Neurological Disorders; Neurotherapeutics; Toxins; Tremor and Other Hyperkinetic Movements; Journal of Parkinsons Disease; UpToDate. ML has received research funding from the Dystonia Medical Research Foundation and Benign Essential Research Foundation. RB is a member of DSC and associated funding for this project. He is director of Botulinum Toxin Clinic and 45% of his effort is performing botulinum toxin injections. FC has received scholarship money from University of Sydney and Honorary from Abbvie. VF receives a salary from NSW Health, has received unrestricted research grants from the Michael J Fox Foundation, Abbvie and Merz, is on Advisory Boards and/or has received travel grants from Abbvie, Allergan, Cavion, Ipsen, Merz, Praxis, Seqirus, Stada, Teva and UCB, and receives royalties from Health Press Ltd. SPi has received funding through the Dystonia Coalition sub-award to University of New Mexico (U54TR18020). AB has received research funding from Merz Pharmaceutical and Allergan. He has also served as a consultant to Allergan. The remaining authors declare that the research was conducted in the absence of any commercial or financial relationships that could be construed as a potential conflict of interest.

## Publisher's Note

All claims expressed in this article are solely those of the authors and do not necessarily represent those of their affiliated organizations, or those of the publisher, the editors and the reviewers. Any product that may be evaluated in this article, or claim that may be made by its manufacturer, is not guaranteed or endorsed by the publisher.

## References

[1] Albanese A Bhatia K Bressman SB Delong MR Fahn S Fung VS. Phenomenology and classification of dystonia: a consensus update. Mov Disord. (2013) 28:863–73. 10.1002/mds.25475 23649720 PMC3729880

[2] Reich SG Factor SA. Therapy of Movement Disorders: A Case-Based Approach. Basel: Springer (2019).

[3] Nutt JG Muenter MD Aronson A Kurland LT Melton LJ 3rd. Epidemiology of focal and generalized dystonia in Rochester, Minnesota. Mov Disord. (1988) 3:188–94. 10.1002/mds.870030302 3264051

[4] Yoshida K. Prevalence and incidence of oromandibular dystonia: an oral and maxillofacial surgery service-based study. Clin Oral Investig. (2021). 10.1007/s00784-021-03878-9. [Epub ahead of print]. 33956216

[5] Defazio G Albanese A Pellicciari R Scaglione CL Esposito M Morgante F. Expert recommendations for diagnosing cervical, oromandibular, limb dystonia. Neurol Sci. (2019) 40:89–95. 10.1007/s10072-018-3586-9 30269178

[6] Jinnah HA Factor SA. Diagnosis and treatment of dystonia. Neurol Clin. (2015) 33:77–100. 10.1016/j.ncl.2014.09.002 25432724 PMC4248237

[7] Jinnah HA. The dystonias. Continuum. (2019) 25:976–1000. 10.1212/CON.0000000000000747 31356290

[8] Esper CD Freeman A Factor SA. Lingual protrusion dystonia: frequency, etiology and botulinum toxin therapy. Parkinsonism Relat Disord. (2010) 16:438–41. 10.1016/j.parkreldis.2010.04.007 20494607

[9] Rosales RL. X-linked dystonia parkinsonism: clinical phenotype, genetics and therapeutics. J Mov Disord. (2010) 3:32–8. 10.14802/jmd.10009 24868378 PMC4027667

[10] Song PC Le H Acuna P De Guzman JK Sharma N Francouer TN. Voice and swallowing dysfunction in X-linked dystonia parkinsonism. Laryngoscope. (2020) 130:171–7. 10.1002/lary.27897 30889292

[11] Scorr LM Silver MR Hanfelt J Sperin E Freeman A Jinnah HA. Pilot single-blind trial of AbobotulinumtoxinA in oromandibular dystonia. Neurotherapeutics. (2018) 15:452–8. 10.1007/s13311-018-0620-9 29542022 PMC5935649

[12] Yoshida K. Botulinum neurotoxin therapy for lingual dystonia using an individualized injection method based on clinical features. Toxins. (2019) 11. 10.3390/toxins11010051 30658420 PMC6357149

[13] LaScorr FS. Treatment of oromandibular dystonia. In: Reich SG Factor S, editors. Therapy of Movement Disorders — A Case Based Approach. Basel: Springer (2019). p. 205–9.

[14] Termsarasab P Tanenbaum DR Frucht SJ. The phenomenology and natural history of idiopathic lower cranial dystonia. J Clin Mov Disord. (2014) 1:3. 10.1186/2054-7072-1-3 26788329 PMC4676493

[15] Comella CL. Systematic review of botulinum toxin treatment for oromandibular dystonia. Toxicon. (2018) 147:96–9. 10.1016/j.toxicon.2018.02.006 29453996

[16] Tan EK Jankovic J. Tardive and idiopathic oromandibular dystonia: a clinical comparison. J Neurol Neurosurg Psychiatry. (2000) 68:186–90. 10.1136/jnnp.68.2.186 10644785 PMC1736782

[17] Kilic-Berkmen G Wright LJ Perlmutter JS Comella C Hallett M Teller J. The dystonia coalition: a multicenter network for clinical and translational studies. Front Neurol. (2021) 12:660909. 10.3389/fneur.2021.660909 33897610 PMC8060489

[18] Ondo WG Simmons JH Shahid MH Hashem V Hunter C Jankovic J. Onabotulinum toxin-A injections for sleep bruxism: a double-blind, placebo-controlled study. Neurology. (2018) 90:e559–64. 10.1212/WNL.0000000000004951 29343468

[19] Schiffman E Ohrbach R Truelove E Look J Anderson G Goulet JP. Orofacial pain special interest group, diagnostic criteria for temporomandibular disorders (DC/TMD) for clinical and research applications: recommendations of the International RDC/TMD Consortium Network^*^ and Orofacial Pain Special Interest Groupdagger. J Oral Facial Pain Headache. (2014) 28:6–27. 10.11607/jop.1151 24482784 PMC4478082

[20] Yoshida K. Oromandibular dystonia screening questionnaire for differential diagnosis. Clin Oral Investig. (2019) 23:405–11. 10.1007/s00784-018-2449-3 29717363

[21] Yan L Hicks M Winslow K Comella C Ludlow C Jinnah HA. Secured web-based video repository for multicenter studies. Parkinsonism Relat Disord. (2015) 21:366–71. 10.1016/j.parkreldis.2015.01.011 25630890 PMC4372455

[22] Patel N Hanfelt J Marsh L Jankovic J members of the Dystonia C. Alleviating manoeuvres (sensory tricks) in cervical dystonia. J Neurol Neurosurg Psychiatry. (2014) 85:882–4. 10.1136/jnnp-2013-307316 24828895 PMC4871143

[23] Blitzer A Brin MF Greene PE Fahn S. Botulinum toxin injection for the treatment of oromandibular dystonia. Ann Otol Rhinol Laryngol. (1989) 98:93–7. 10.1177/000348948909800202 2916831

[24] Jankovic J Schwartz K Donovan DT. Botulinum toxin treatment of cranial-cervical dystonia, spasmodic dysphonia, other focal dystonias and hemifacial spasm. J Neurol Neurosurg Psychiatry. (1990) 53:633–9. 10.1136/jnnp.53.8.633 2213039 PMC488162

[25] Hermanowicz N Truong DD. Treatment of oromandibular dystonia with botulinum toxin. Laryngoscope. (1991) 101:1216–8. 10.1288/00005537-199111000-00010 1943423

[26] Van den Bergh P Francart J Mourin S Kollmann P Laterre EC. Five-year experience in the treatment of focal movement disorders with low-dose Dysport botulinum toxin. Muscle Nerve. (1995) 18:720–9. 10.1002/mus.880180708 7783762

[27] Sankhla C Lai EC Jankovic J. Peripherally induced oromandibular dystonia. J Neurol Neurosurg Psychiatry. (1998) 65:722–8. 10.1136/jnnp.65.5.722 9810945 PMC2170345

[28] Tan EK Jankovic J. Botulinum toxin A in patients with oromandibular dystonia: long-term follow-up. Neurology. (1999) 53:2102–7. 10.1212/WNL.53.9.2102 10599789

[29] Bhattacharyya N Tarsy D. Impact on quality of life of botulinum toxin treatments for spasmodic dysphonia and oromandibular dystonia. Archives of otolaryngology–head & neck surgery. (2001) 127:389–92. 10.1001/archotol.127.4.389 11296046

[30] Adler CH Factor SA Brin M Sethi KD. Secondary nonresponsiveness to botulinum toxin type A in patients with oromandibular dystonia. Mov Disord. (2002) 17:158–61. 10.1002/mds.10001 11835455

[31] Lo SE Rosengart AJ Novakovic RL Kang UJ Shah DN Khan MA. Identification and treatment of cervical and oromandibular dystonia in acutely brain-injured patients. Neurocrit Care. (2005) 3:139–45. 10.1385/NCC:3:2:139 16174883

[32] Wan XH Vuong KD Jankovic J. Clinical application of botulinum toxin type B in movement disorders and autonomic symptoms. Chin Med Sci J. (2005) 20:44–7. 15844312

[33] Singer C Papapetropoulos S. A comparison of jaw-closing and jaw-opening idiopathic oromandibular dystonia. Parkinsonism Relat Disord. (2006) 12:115–8. 10.1016/j.parkreldis.2005.07.007 16271495

[34] Lee KH. Oromandibular dystonia. Oral Surg Oral Med Oral Pathol Oral Radiol Endod. (2007) 104:491–6. 10.1016/j.tripleo.2007.04.001 17689275

[35] Merz RI Deakin J Hawthorne MR. Oromandibular dystonia questionnaire (OMDQ-25): a valid and reliable instrument for measuring health-related quality of life. Clin Otolaryngol. (2010) 35:390–6. 10.1111/j.1749-4486.2010.02194.x 21108749

[36] Teive HA Kluppel LE Munhoz RP Becker N Muller PR Werneck LC. Jaw-opening oromandibular dystonia secondary to Wilson's disease treated with botulinum toxin type A. Arq Neuropsiquiatr. (2012) 70:407–9. 10.1590/S0004-282X2012000600005 22699536

[37] Sinclair CF Gurey LE Blitzer A. Oromandibular dystonia: long-term management with botulinum toxin. Laryngoscope. (2013) 123:3078–83. 10.1002/lary.23265 24122897

[38] Bakke M Larsen BM Dalager T Moller E. Oromandibular dystonia–functional and clinical characteristics: a report on 21 cases. Oral Surg Oral Med Oral Pathol Oral Radiol. (2013) 115:e21–6. 10.1016/j.oooo.2012.04.023 22999966

[39] Moscovich M Chen ZP Rodriguez R. Successful treatment of open jaw and jaw deviation dystonia with botulinum toxin using a simple intraoral approach. J Clin Neurosci. (2015) 22:594–6. 10.1016/j.jocn.2014.08.027 25541097

[40] Teemul TA Patel R Kanatas A Carter LM. Management of oromandibular dystonia with botulinum A toxin: a series of cases. Br J Oral Maxillofac Surg. (2016) 54:1080–4. 10.1016/j.bjoms.2016.06.028 27776922

[41] Nastasi L Mostile G Nicoletti A Zappia M Reggio E Catania S. Effect of botulinum toxin treatment on quality of life in patients with isolated lingual dystonia and oromandibular dystonia affecting the tongue. J Neurol. (2016) 263:1702–8. 10.1007/s00415-016-8185-1 27278063

[42] Kreisler A Verpraet AC Veit S Pennel-Ployart O Behal H Duhamel A. Clinical characteristics of voice, speech, and swallowing disorders in oromandibular dystonia. J Speech Lang Hear Res. (2016) 59:940–9. 10.1044/2016_JSLHR-S-15-0169 27617622

[43] Slaim L Cohen M Klap P Vidailhet M Perrin A Brasnu D. Oromandibular dystonia: demographics and clinical data from 240 patients. J Mov Disord. (2018) 11:78–81. 10.14802/jmd.17065 29860784 PMC5990905

[44] Wang YP Gorenstein C. Psychometric properties of the Beck Depression Inventory-II: a comprehensive review. Braz J Psychiatry. (2013) 35:416–31. 10.1590/1516-4446-2012-1048 24402217

[45] Mennin DS Fresco DM Heimberg RG Schneier FR Davies SO Liebowitz MR. Screening for social anxiety disorder in the clinical setting: using the Liebowitz Social Anxiety Scale. J Anxiety Disord. (2002) 16:661–73. 10.1016/S0887-6185(02)00134-2 12405524

[46] Kojovic M Parees I Kassavetis P Palomar FJ Mir P Teo JT. Secondary and primary dystonia: pathophysiological differences. Brain. (2013) 136:2038–49. 10.1093/brain/awt150 23771342

[47] Schneider SA Bhatia KP. Secondary dystonia-clinical clues and syndromic associations. J Mov Disord. (2009) 2:58–63. 10.14802/jmd.09016 24868358 PMC4027713

[48] Ortiz R Scheperjans F Mertsalmi T Pekkonen E. The prevalence of adult-onset isolated dystonia in Finland 2007-2016. PLoS ONE. (2018) 13:e0207729. 10.1371/journal.pone.0207729 30458031 PMC6245745

[49] O'Riordan S Raymond D Lynch T Saunders-Pullman R Bressman SB Daly L. Age at onset as a factor in determining the phenotype of primary torsion dystonia. Neurology. (2004) 63:1423–6. 10.1212/01.WNL.0000142035.26034.C2 15505159

[50] Jinnah HA Berardelli A Comella C Defazio G Delong MR Factor S. Dystonia Coalition, the focal dystonias: current views and challenges for future research. Mov Disord. (2013) 28:926–43. 10.1002/mds.25567 23893450 PMC3733486

[51] Friedman A Fahn S. Spontaneous remissions in spasmodic torticollis. Neurology. (1986) 36:398–400. 10.1212/WNL.36.3.398 3951708

[52] Yoshida K. Development and validation of a disease-specific oromandibular dystonia rating scale (OMDRS). Front Neurol. (2020) 11:583177. 10.3389/fneur.2020.583177 33224096 PMC7669987

[53] Girach A Vinagre Aragon A Zis P. Quality of life in idiopathic dystonia: a systematic review. J Neurol. (2019) 266:2897–906. 10.1007/s00415-018-9119-x 30460447 PMC6851210

[54] Ma H Qu J Ye L Shu Y Qu Q. Blepharospasm, oromandibular dystonia, and meige syndrome: clinical and genetic update. Front Neurol. (2021) 12:630221. 10.3389/fneur.2021.630221 33854473 PMC8039296

[55] Jinnah HA. Medical and surgical treatments for dystonia. Neurol Clin. (2020) 38:325–48. 10.1016/j.ncl.2020.01.003 32279713 PMC7156436

[56] Wang X Zhang Z Mao Z Yu X. Deep brain stimulation for Meige syndrome: a meta-analysis with individual patient data. J Neurol. (2019) 266:2646–56. 10.1007/s00415-019-09462-2 31302747

